# Postharvest Reduction of *Salmonella enterica* on Tomatoes Using a Pelargonic Acid Emulsion

**DOI:** 10.3390/foods10010178

**Published:** 2021-01-17

**Authors:** Elizabeth White, Govindaraj Dev Kumar, Andre Luiz Biscaia Ribeiro da Silva, William L. Kerr, Samuel Cimowsky, J. Andrew Widmer, Laurel L. Dunn

**Affiliations:** 1 Department of Food Science & Technology, University of Georgia, 100 Cedar St., Athens, GA 30602, USA; elizabeth.white26@uga.edu (E.W.); wlkerr@uga.edu (W.L.K.); samuel.cimowsky25@uga.edu (S.C.); james.widmer@uga.edu (J.A.W.); 2 Center for Food Safety, University of Georgia, 1109 Experiment St, Griffin, GA 30223, USA; 3 Department of Horticulture, University of Georgia, 4606 Research Way, Tifton, GA 31793, USA; adasilva@uga.ed

**Keywords:** produce safety, postharvest wash, sanitizer, chlorine, peroxyacetic acid

## Abstract

A novel produce wash consisting of pelargonic acid (PEL) emulsions was tested on tomatoes contaminated with a five-serovar *Salmonella enterica* cocktail. Ability to reduce contamination on the inoculated tomato surface, as well as mitigation of subsequent cross-contamination to uninoculated tomatoes washed in re-used/spent wash water were examined. Sanitizer efficacy was also examined over 1 and 7 d storage time (8 °C, recommended for red ripe tomatoes) and in the presence of 0.5% (*w*/*v*) organic load. PEL performed statistically the same (*p* ≤ 0.05) at both 30 mM and 50 mM concentrations and resulted in greater than 1, 5 and 6 log CFU/g *Salmonella* reductions at 0 h, 1 d and 7 d, respectively, when compared to a water-only or no rinse (NR) treatment. This was also a significantly greater reduction than was observed due to chlorine (sodium hypochlorite) and peroxyacetic acid (PAA) at all time points (*p* ≤ 0.01). Organic load had no impact on sanitizer efficacy for all examined treatments. Finally, PEL had a deleterious impact on tomato texture. At 1 d, ca. 5 N and 7 N were required to achieve tomato skin penetration and compression, respectively, compared to >9 N and 15 N required by all other treatments (*p* ≤ 0.05). While PEL sanitizers effectively reduced inoculated *Salmonella* and subsequent transfer to uninoculated tomatoes, reformulation may be necessary to prevent deleterious quality impacts on produce.

## 1. Introduction

Illnesses associated with foodborne pathogens affect 48 million people in the United States each year [1]. Of those sickened, 128,000 are hospitalized and 3000 die [1,2,3] indicating that foodborne illnesses could often result in severe consequences to public health. *Salmonella enterica* subspecies *enterica* is the leading cause of bacterial foodborne outbreaks associated with produce and the leading cause of hospitalizations and deaths related to foodborne diseases in the U.S. [3,4]. *Salmonella* outbreaks were historically associated with foods of animal origin; however, since 2010, tomatoes and other produce types have been linked to over 75 salmonellosis outbreaks throughout the U.S. [5,6]. From 1990 to 2007, approximately 2000 culture-confirmed cases from 12 multistate outbreaks of salmonellosis were traced back to tomatoes [6].

In 2004, three tomato-associated outbreaks occurred in North America resulting in a total of 561 illnesses [7]. The largest of the three was traced back to Roma tomatoes in convenience store sandwiches, causing 429 illnesses and involving the serovars Javiana, Typhimurium, Anatum, Thompson and Munchen [7]. The other two outbreaks were caused by individual serotypes; a *S.* Braenderup outbreak impacted people in 16 states and caused 125 illnesses, whereas the other outbreak sickened seven people in one Canadian province and was caused by *S.* Javiana [7]. Additionally, *S. enterica* serovars Saintpaul, Enteriditis, Hartford, Typhimurium and Berta have also been implicated in tomato-related salmonellosis outbreaks since 2004 [8].

While good agricultural practices (GAPs) are critical to limit initial contamination of produce, postharvest sanitizers can reduce microbial cross-contamination and internalization in the event contaminated produce is introduced to postharvest wash tanks. The Produce Safety Rule (PSR) does not require the use of sanitizers during postharvest washing [9], but the North American Tomato Trade Work Group and United Fresh Produce Association’s Commodity Specific Food Safety Guidelines for the Fresh Tomato Supply Chain recommend chlorine or other U.S. Environmental Protection Agency (EPA)-registered antimicrobial pesticides approved for use in postharvest wash water to control cross-contamination [10,11].

Chlorine and peroxyacetic acid (PAA) are both strong oxidizers and two of the most frequently used sanitizers for postharvest washing [12,13]. Chlorine, the more economical option of the two, requires robust monitoring and pH adjustment as fluctuations in pH, temperature, organic matter and other factors interfere with its efficacy [12,13,14,15]. PAA is less impacted by extrinsic factors and does not require pH adjustment but is generally more expensive than chlorine [12,13]. Both sanitizers are also highly corrosive and may pose a worker health hazard when mishandled [16]. Additionally, while chlorine and PAA can result in 4 to 5 log CFU/mL reductions of foodborne pathogens within the wash system [17], effectively reducing cross-contamination, they typically result in <1 to 2.5 log CFU/g reductions on the produce or sprout surface [14,18,19,20]. For these reasons, alternative mitigation strategies are of increasing interest to produce growers and researchers alike.

One such novel, alternative sanitizer being examined is pelargonic acid (PEL). PEL, also known as nonanoic acid, is a nine-carbon saturated fatty acid naturally occurring in plants, including tomatoes and animals [21,22,23,24,25]. Pelargonic acid is of interest as a sanitizer because it exhibits both antibacterial and antifungal activity [23,26,27]. The primary mode of antimicrobial action is membrane disruption resulting in eventual cell lysis [22,28]. A study of PEL and its derivatives found that PEL was inhibitory to several outbreak and environmental isolates of *Salmonella* [21,23]. Additionally, organic acids, including PEL, are usually stable in the presence of organic matter, such as is encountered to varied degrees in produce wash systems [12]. PEL is registered with the EPA [29], is nontoxic to humans in small quantities [28] and as of 2003, is exempt from the requirement of a tolerance of residues in or on all foods when applied as a component of a food contact surface sanitizing solution in food handling [30]. However, it is not currently validated or approved for use in produce wash water.

The following study had three objectives. First, the antimicrobial efficacy of two PEL emulsions against a *Salmonella* cocktail inoculated onto the tomato surface was examined and compared to the efficacy of free chlorine (sodium hypochlorite) and PAA. Sanitizer control of wash water-mediated cross-contamination from inoculated to uninoculated tomatoes submerged in the same, spent wash solution was also quantified. Finally, the impact of the PEL emulsion on tomato texture was evaluated and compared to chlorine, PAA, water and no rinse (NR) treatments.

## 2. Materials and Methods 

### 2.1. Bacterial Cultures

A five-serovar cocktail of *Salmonella enterica* subspecies *enterica* was used: *S. enterica* Agona (alfalfa sprout-associated outbreak), Saintpaul (tomato/pepper-associated outbreak), Newport (environmental isolate, tomato-associated outbreak), Montevideo (clinical isolate, tomato-associated outbreak) and Kentucky (poultry litter isolate). Serovars Agona, Saintpaul, Newport and Montevideo were acquired from the Department of Food Science and Technology culture collection at the University of Georgia and serovar Kentucky was provided by Dr. Michelle Danyluk at the University of Florida. All cultures were serologically confirmed using latex agglutination (Oxoid, Ogdensberg, NY, USA) prior to experimentation.

All *Salmonella* strains were stepwise adapted to 40 ppm nalidixic acid (Alfa Aesar, Haverhill, MA, USA). Preliminary findings (not shown) demonstrated that the nalidixic acid alone did not effectively prevent growth of the grape tomato background microflora, so the strains were additionally adapted to 40 ppm rifampicin (Research Products International, Mount Prospect, IL, USA). Stepwise adaptation was performed in tryptic soy broth (TSB; BD Difco, Sparks, MD, USA) with antibiotic concentrations increasing each subsequent transfer in 5 to 10 ppm increments. 

Prior to use, individual, antibiotic-adapted cultures were transferred from frozen glycerol stocks into TSB containing 40 ppm nalidixic acid and 40 ppm rifampicin (TSBRN), incubated for 24 h at 37 °C and transferred (10 μL) into fresh TSBRN for two subsequent times. A 300 μL aliquot of each pure culture was individually spread plated onto tryptic soy agar plates (BD Difco, Sparks, MD, USA) containing 40 ppm NA and 40 ppm rifampicin (TSARN), then incubated for 20 h at 37 °C to create a bacterial lawn. Plate surfaces were scraped with sterile L-shaped spreaders (Thermo Fisher Scientific, Waltham, MA, USA) and rinsed with 5 mL of sterile phosphate-buffered saline (PBS, pH 7.2; Thermo Fisher Scientific, Waltham, MA, USA) to harvest bacterial cultures; each serotype was combined in equal volumes into a 50 mL conical centrifuge tube to form the *Salmonella* cocktail used for inoculation. The culture was centrifuged (Centrifuge 5810, Eppendorf, Hamburg, Germany) at 2300× *g* for 15 min, the supernatant was decanted and the pellet was vortexed to resuspend in fresh PBS. The cocktail was serially diluted and enumerated using the Eddy Jet 2 spiral plater (Neutec, Farmingdale, NY, USA) on TSARN plates to verify that the concentration of the final inoculum was ca. 10 log CFU *Salmonella*/mL.

### 2.2. Tomato Inoculation 

Each sample, comprised of two ripe, organic grape tomatoes (*Solanum* sp.), was spot inoculated using 10 μL drops of culture. Culture was placed on the tomato cheek avoiding the stem and blossom scars until a final volume of 200 μL/sample (20 drops, 10 per tomato) was reached. Inoculated samples were placed in the biosafety cabinet and allowed to dry for 2 h. Four replicates of two samples each (*n* = 8; 16 tomatoes per replicate) were examined for each sanitizer, sanitizer plus OL or control.

### 2.3. Emulsion Preparation

A 1 M stock emulsion of n-Pelargonic acid, 97% (Pelargonic acid, Acros Organics, NJ, USA) in water was prepared using saponin from quillaja bark (Sigma-Aldrich, St. Louis, MO, USA) at a concentration of 0.1% (*w*/*v*) as described by Kumar et al. [23]. The emulsion was formed by stirring the pelargonic acid-quillaja saponin mixture at 1100 rpm (Isotemp™ Hot Plate, Fisher Scientific, Pittsburgh, PA, USA) at a temperature of 25°C for 10 min. The 1 M PEL and quillaja saponin emulsion was then diluted with sterile deionized water to achieve final concentrations of 30 mM PEL and 50 mM PEL.

### 2.4. Preparation of Wash Systems

Treatments were tested against *Salmonella* with and without 0.5% (*w*/*v*) organic load (OL). The OL was made by pureeing grape tomatoes and then adding the puree to the wash treatments. The treatments of 200 ppm free chlorine, 80 ppm PAA, 30 mM PEL and 50 mM PEL were each tested with and without added OL; water and NR controls were examined without OL. Autoclave-sterilized deionized water was also examined to simulate washing without a sanitizer present. All treatment rinses were prepared in 500 mL volumes. The chlorine sanitizer was made by adding Clorox Regular Bleach (The Clorox Company, Oakland, CA, USA; EPA registration number 5813-1) to sterile, deionized water to a free chlorine concentration of 200 ppm, which was verified with an Ultra High Range Chlorine Portable Photometer (Hanna Instruments, Woonsocket, RI, USA) immediately prior to use. The pH of the chlorine solution was also adjusted to 7.0 (±0.02) using 1 M citric acid. PAA solution was made by mixing Sanidate 5.0 (BioSafe Systems, East Hartford, CT, USA) in sterile, deionized water. The final concentration of 80 ppm PAA was verified using a PAA Test Kit (TSCTK7500-Z, Thomas Scientific, Swedesboro, NJ, USA). To simulate field packing, a NR control was also examined; this evaluated the level of *Salmonella* inoculated onto tomatoes that received no treatment. A sterile water control was used to simulate washing without a sanitizer in the system. The pH values of each treatment solution with and without organic load were measured immediately prior to the introduction of inoculated tomatoes (Accumet AB250 pH/ISE meter, Thermo Fisher Scientific, Waltham, MA, USA) to determine the extent to which the acidic tomato puree (OL) affected the pH of the solutions (Table 1).

### 2.5. Simulated Postharvest Treatment of Inoculated Samples

*Salmonella* was enumerated from inoculated grape tomatoes that received no treatment (NR) to determine the initial inoculum for each replicate. Two inoculated tomatoes were submerged for 2 min without agitation in a glass beaker containing 500 mL of the wash solution. After 2 min, tomatoes were removed from the wash solution with sterile tongs, placed into a stomacher bag and diluted 1:5 (*w*/*v*) in phosphate buffer solution (PBS) with 0.2% Tween 80 (Sigma-Aldrich, St. Louis, MO, USA) and 0.1% sodium thiosulfate (Sigma-Aldrich, St. Louis, MO, USA). Diluted samples were hand massaged for 20 s; the rinsate was then serially diluted in buffered peptone water (BPW; BD Difco, Sparks, MD, USA). Samples were spiral plated (Eddy Jet 2 spiral plater, Neutec, Farmingdale, NY, USA) onto TSARN. The TSARN plates were incubated 24 h at 37 °C, after which time colonies were counted.

### 2.6. Cross-Contamination to Uninoculated Samples

Immediately after the inoculated tomatoes were removed from the wash treatment, a subsequent, uninoculated sample (SUS 1) comprised of two tomatoes was washed in the same, used wash solution in order to evaluate the degree of cross-contamination from the initial, contaminated sample. The SUS 1 sample was submerged for 2 min, removed and a second SUS sample (SUS 2) was added to the same, used solution for 2 min treatment. Immediately upon removal from solution, SUS 1 and SUS 2 samples were diluted 1:5 (*w*/*v*) in PBS with 0.2% Tween 80 and 0.1% sodium thiosulfate, hand massaged for 20 s and spiral plated. A 10 mL aliquot of the used rinsate was also vacuum filtered through a 0.45-μm mixed cellulose ester membrane filter type HA (GE Healthcare Bio-Sciences, Piscataway, NJ, USA) to quantify residual *Salmonella* in the wash water. The filter was placed grid-side up on TSARN, incubated for 24 h at 37 °C and colonies were counted. 

### 2.7. Simulated Storage and Examination 

Additional inoculated tomatoes and SUS samples (1 and 2) were washed as previously described but instead of undergoing immediate enumeration were stored for either 1 d or 7 d. At each time point, all treatment washes and NR were replicated four times with two samples per replicate (*n* = 8) using the previously described method. Because the samples were at the red ripe stage (USDA color stage 6), tomatoes were stored for either 1 or 7 d at the recommended 8 °C, while relative humidity (RH) was held at 70% [31,32]. After storage, tomato samples were diluted, hand massaged and plated as previously described. Ten mL of rinsate from inoculated samples and SUS was also filtered and incubated as previously described.

### 2.8. Texture Analyses 

Uninoculated organic grape tomatoes were washed in all treatment solutions, including NR and stored at 8 °C and 70% RH for 0 d, 1 d or 7 d prior to texture analysis. All treatments were replicated four times with two samples per replicate (*n* = 8) at 0 d, 1 d and 7 d time points.

After treatment and respective storage, tomato texture was analyzed using both penetration (skin strength) and compression (tomato firmness) tests.

As adapted from Pinheiro et al. [33], tomato skin strength was determined by a penetration test with a TA-XT2 texture analyzer (Stable Micro Systems, Godalming, UK), using a 50 kg load cell and a stainless-steel cylinder probe with a 3 mm diameter. The penetration test was performed at a speed of 3 mm/s and 7.5 mm of penetration distance at the equatorial zone of the fruits. Maximum peak force (N) was used as a measure of the fruit penetration force. It is important to note that this test subjects the sample to both shear and compressive forces so it can detect when the skin ruptures.

The TA-XT2 texture analyzer was also used for a compression test, adapted from Arazuri et al. [34]. A stainless-steel circular flat plate attachment (45 mm diameter) compressed the sample at 1 mm/s to a distance of 5 mm in the equatorial zone. Maximum force (N) needed to compress 5 mm of the tomato in the equatorial zone was recorded as the firmness.

### 2.9. Statistical Analysis

Statistical analyses were performed using generalized linear mixed techniques in the R Studio (RStudio Team, 2016). Inoculated samples were analyzed with sanitizer (i.e., chlorine, PAA, 30 mM PEL and 50 mM PEL), organic load (i.e., presence of organic load or not), sampling timing (i.e., 0, 1 and 7 d) and their interactions as fixed effects. The two SUS were analyzed with sanitizer, organic load, sampling timing, SUS (1 and 2) and their interactions as fixed effects. In both models, sampling time was treated as a repeated measurement and the heterogeneous compound symmetry was used as the covariance structure due its smallest Akaike’s information. Wash solution was analyzed with sanitizer, organic load and their interactions as fixed effects. There were no significant third or second order interactions among OL, sanitizer and storage duration treatments for *Salmonella*, except for the interaction of sanitizer and storage duration; because of this, OL and non-OL samples for chlorine, PAA and both 30 and 50 mM PEL sanitizers were combined and analyzed together (*n* = 16). Because water and NR had no OL equivalent, the sample size remained at *n* = 8. For all analyzes, when the F value was significant least square means comparisons were performed using the Tukey adjusted probability value of 0.05 and means were portioned as needed. Additionally, orthogonal contrasts were used to evaluate the effect of sanitizer versus water or NR. In addition, analysis was done on data from water versus NR for inoculated samples, as well as that for sanitizer versus water for SUS and wash solution.

Penetration and compression of tomato fruit were analyzed with sanitizer plus water and NR, sampling time and their interactions as fixed effects. Once again, sampling timing was treated as a repeated measurement and the heterogeneous compound symmetry was used as the covariance structure due its smallest Akaike’s information. When the F value was significant, least square means comparisons were performed using the Tukey adjusted probability value of 0.05 and means were portioned as needed.

## 3. Results

### 3.1. Reduction of Salmonella on Inoculated Tomatoes Over Time 

Overall, *Salmonella* counts on tomatoes treated with PEL decreased to a greater extent than *Salmonella* on tomatoes treated with chlorine, PAA, water or NR. The PEL emulsions also controlled cross-contamination onto SUS significantly better than chlorine and PAA. Overall, sanitizer type and storage time significantly interacted with *Salmonella* populations (Table 2) but organic load had no significant impact on sanitizer efficacy at any time point.

At 0 d, both PEL emulsions resulted in significant reductions (ca. 1 log CFU/g) over chlorine and PAA (Table 2). Chlorine (7.03 log CFU/g) and PAA (6.73 log CFU/g) performed statistically similarly to the water (7.35 log CFU/g) and NR (7.53 log CFU/g) controls, while both PEL emulsions resulted in significantly greater reductions than chlorine, PAA, water and NR (*p* ≤ 0.05).

After 1 d storage, all four sanitizers resulted in significantly greater *Salmonella* reductions than the water and NR controls. Chlorine caused a significantly lower reduction than all other sanitizers at 1 d, with 5.38 log CFU/g *Salmonella* recovered. PAA (3.23 log CFU/g) performed significantly better than chlorine at 1 d but was less effective than both PEL sanitizers, from which 1.86 log CFU/g (30 mM PEL) and 1.16 log CFU/g (50 mM PEL) *Salmonella* were recovered. By comparison, *Salmonella* was recovered at 7.59 and 7.96 log CFU/g from water and NR, respectively, after 1 d storage.

A similar trend occurred after 7 d. Chlorine (5.28 log CFU/g) reduced recoverable *Salmonella* better than water (7.57 log CFU/g) and NR (7.73 log CFU/g) controls but not as well as PAA (2.92 log CFU/g), which in turn was less effective than 30 mM PEL (1.00 log CFU/g) and 50 mM PEL (1.15 log CFU/g). All sanitizers at 1 and 7 d performed better than their 0 d counterpart.

### 3.2. Cross-Contamination on SUS Tomatoes

Water did not limit *Salmonella* cross-contamination onto the SUS. At 0 d, water SUS 1 and SUS 2 had mean *Salmonella* populations of 4.1 ± 0.5 and 4.1 ± 0.8 log CFU/g, respectively. From 0 d to 7 d, mean *Salmonella* populations on water SUS 1 and SUS 2 decreased by 0.5 ± 0.9 and 0.4 ± 1.0 log CFU/g, respectively, leaving viable *Salmonella* populations on 7 d SUS (1 and 2) less than 3.5 log CFU/g. For each sanitizer, mean *Salmonella* recovered from both SUS 1 and SUS 2 was below 1.1 log CFU/g at all time points (i.e., 0 d, 1 d, 7 d); the PEL treatments reduced *Salmonella* to below the limit of detection (1 log CFU/g) at all time points. There was no significant difference in the mean log CFU/g of SUS 1 and SUS 2 within any of the respective treatments or time points.

After the treatment of the inoculated tomatoes and two SUS, *Salmonella* was present at a mean of 5.7 ± 0.4 log CFU/mL in the water wash solution. *Salmonella* was not detectable in any of the wash treatments that contained sanitizers (limit of detection: −1 log CFU/mL).

### 3.3. Effect of Sanitizers on Fruit Texture Over Time

At 0 d, there was no significant difference in tomato skin strength (measured by penetration) among the four sanitizers or water (*p* ≥ 0.05; Table 3).

The 50 mM PEL was the only treatment for which skin strength at 0 d was significantly lower than that of NR (*p* ≤ 0.05; Table 3). Skin strength at 0 d ranged from 7.23 (50 mM PEL) to 9.82 N (NR). There were no significant differences in skin strength between NR, water, chlorine or PAA at 0, 1 or 7 d. However, the skin strength of tomatoes treated with 30 mM or 50 mM PEL decreased by 3.83 and 2.62 N, respectively, from 0 d to 1 d. No treatments exhibited significant differences in tomato skin strength between 1 d and 7 d. Skin strength at 0 d and 7 d were significantly different for both 30 mM and 50 mM PEL (mean decrease: 4.57 and 3.72 N, respectively).

At 0 d, there was no significant difference in firmness measured by compression among the four sanitizers, water or NR (*p* ≤ 0.05), with an average firmness of 16.15 N (Table 4).

The 30 mM and 50 mM PEL treatments were the only ones for which there was a significant difference in firmness from 0 d to 1 d. For example, the maximum force of the 50 mM PEL sample decreased from 15.30 N at 0 d to 7.17 N at 1 d. NR, water and PAA exhibited significant increases in firmness from 1 d to 7 d. Chlorine was the only treatment for which no significant change in tomato firmness occurred over time.

## 4. Discussion

Overall, the PEL sanitizer treatments significantly reduced *Salmonella* recovery from tomatoes, both immediately post-treatment and after storage. *Salmonella* was recovered at a rate of 5.76 log CFU/g (PEL 30) and 5.37 log CFU/g (PEL 50) immediately post-treatment, an improvement of 1.77 and 2.16 log CFU/g over simulated field packed (NR control) tomatoes. The least effective sanitizers examined, chlorine (7.03 log CFU/g) and PAA (6.73 log CFU/g), resulted in statistically similar reductions of *Salmonella* at 0 d. These reductions were also statistically similar to those observed by the water (7.35 log CFU/g) and NR (7.53 log CFU/g) controls. In a 2019 study, Dunn et al. found similar reductions on *Salmonella*-inoculated peppers; 200 ppm chlorine resulted in a statistically significant but less than 1 log CFU/g reduction when compared to a NR rinse control and performed statistically the same as the water control [14]. Interestingly, Luo et al. found that a 200-ppm wash used on *Salmonella* inoculated grape tomatoes resulted in a greater than 4 log CFU/g reduction. However, the tomatoes in the Wu and Lu study were rinsed under continuous agitation for 5 or 10 min [35], which may be more comparable to conditions and exposure time in a hydrocooler [36]. The current study’s 2 min treatment in a static bath was intended to mimic typical exposure time in a packinghouse dump tank.

The most significant differences in antimicrobial efficacy among treatments occurred during storage. Throughout storage (0 d to 7 d), *Salmonella* populations on inoculated NR and water-washed control tomatoes water underwent no significant changes and were statistically similar to each other. This is consistent with the findings of Zhuang et al. [37], which found that populations of *S. enterica* Montevideo inoculated onto the surface of mature green tomatoes remained relatively constant when stored at 10 °C for 18 d. Survival during this extended period indicates that *Salmonella* can survive throughout tomato transport in the absence of mitigation strategies (e.g., sanitizing washes) and could remain viable when it eventually reaches the consumer. Conversely, both PEL treatments resulted in significant decreases (>3.8 log CFU/g) in *Salmonella* from 0 d to 1 d, after which populations persisted at very low levels, close to or below the limit of detection of 1 log CFU/g through 7 d storage. The antibacterial efficacy of chlorine was most evident from 0 d to 1 d, when it resulted in a statistically significant, nearly 2 log CFU/g reduction (7.03 log CFU/g to 5.38 log CFU/g); however, no significant, continued biocidal activity occurred from 1 d to 7 d (5.29 log CFU/g). The PAA treated tomatoes over time performed significantly better than chlorine at 1 d (3.23 log CFU/g *Salmonella* recovered) but was a less robust biocide than both PEL treatments. This trend continued to 7 d, at which time 2.92 log CFU/g *Salmonella* was recovered from PAA-treated tomatoes.

The storage results indicate that PEL emulsions may be useful biocidal agents, especially on commodities or surfaces where bacterial regrowth or post-processing contamination is anticipated. Produce may be particularly susceptible to contamination while in transit to retailers or restaurants compared to most ready-to-eat foods because it is typically stored and shipped in vented, unsealed containers to facilitate gas exchange due to continued, postharvest metabolic activity [38]. While many of these containers (e.g., clamshells for blueberries, vented bags for grapes, etc.) are shipped in secondary boxes or crates, these too are vented by design and may allow environmental microbial hazards contact to produce contained within. Sanitizers that exhibit continued microbial control for days after initial washing could be useful to protect against contamination while in transit and could be invaluable to control endemic spoilage microorganisms present on the produce surface. Interestingly, PAA also appeared to exert continued antimicrobial activity throughout storage. While the reduction from 1 d to 7 d was not significantly different, from 0 d to 7 d PAA resulted in a significant, nearly 4 log CFU/g reduction; this was a 2 log CFU/g greater reduction at 7 d compared to chlorine at 7 d. While generally a more expensive sanitizer, PAA does not require stringent pH monitoring and adjustment such as is required in wash systems using chlorine, making it easier to incorporate into a produce wash system. The enhanced antimicrobial efficacy over time, its relative ease of use compared to chlorine and degradation into nontoxic compounds is a key consideration for growers as they select or change postharvest sanitizers [13].

One possible explanation for the lack of significant *Salmonella* reduction during storage after treatment with PAA or chlorine is that rifampicin and nalidixic acid-adapted *Salmonella* could have had a cross-tolerance to the oxidizers. Examples of previously described development of antimicrobial cross-tolerances include *Listeria monocytogenes* grown under acidic conditions exhibiting increased tolerance to sodium dichloroisocyanuric acid and didecyl dimethyl ammonium bromide (DDAB) and *S. enterica* ser. Typhimurium grown in the presence of plant-derived terpenes exhibiting increased tolerance to PAA and DDAB [39,40]. Examining wild-type strains and recovering on *Salmonella*-specific media (i.e., Xylose Lysine Deoxycholate agar; XLD) may have reduced any interference due to cross-tolerance but preliminary studies (data not shown) indicated that XLD insufficiently reduced background microflora from the tomato surface.

The ability of each sanitizer to prevent cross-contamination was also investigated. Very low populations of *Salmonella* (<1.0 log CFU/g) were recovered from SUS exposed to sanitizers. This is consistent with the results of the wash solution sampling, as no *Salmonella* was recovered from sanitizers. Conversely, 4.1 log CFU/g was recovered from SUS exposed to water (0 d) and 5.7 log CFU/mL were recovered from spent water after it was used to wash the inoculated tomatoes and SUS. These results show that the four sanitizers were similarly effective to each other and also superior to water in ability to limit cross-contamination. These findings agree with previous research showing that solutions containing sanitizers including chlorine and PAA are more effective than water alone at maintaining microbial quality of postharvest wash tanks [14,37,41,42].

Because the 30 mM formulation performed similarly to 50 mM, lower PEL concentrations should be examined to determine the minimum range required for equivalent biocidal activity. Kumar and Micallef [22] used a modified resazurin assay to determine that the minimal inhibitory concentration (MIC) of the PEL emulsion against *Salmonella* in a 96-well plate was 31.25 mM. However, the MIC could differ when PEL is added to a food matrix, so concentrations below 30 mM may still be viable options. Kumar and Micallef also found that PEL caused a decrease in culturable *Salmonella* cells within one hour, however live-dead staining revealed a mixture of viable and dead cells [22]. Survival of such stress could lead to resistance to PEL and cross-tolerances to other environmental stressors such as heat, starvation and other sanitizers. Once genetic encoding for resistance occurs, it is mobile within a population and has the potential to be horizontally transferred to other populations [43].

A 2012 study found that *S. enterica* ser. Typhimurium can form biofilms on the surface of cherry tomatoes after 48 h of exposure at 25 °C, which has been visualized using scanning electron microscopy [44]. Biofilms increase bacterial resistance to oxidative stress [45], which may impact the efficacy of oxidative sanitizers like chlorine and PAA. However, PEL treatments form an oily coating on the tomato and other surfaces, potentially prolonging PEL contact time with the fruit surface and reducing the ability of *Salmonella* to effectively attach to the tomato surface and subsequently form biofilms. This biocidal activity could also reduce the likelihood of subsequent tomato contamination during further handling or while in transit. However, as PEL is phytotoxic, this increased contact time may contribute a deleterious impact on tomato firmness and skin strength over time when compared to chlorine and PAA-treated tomatoes.

Undissociated organic acids penetrate the phospholipid membrane of bacterial cells, then dissociate once inside of the cell. The dissociation into anion and proton acidifies the internal cellular pH, requiring the cell to expend energy to maintain a roughly neutral pH [43,46]. Below its pKa of 4.95, PEL is predominantly present in undissociated form and is biocidal; however, if the pH of postharvest wash water increases above 4.95, PEL dissociates and loses antimicrobial efficacy [12,47]. In this study, the average pH of the PEL treatments ranged from 3.89 ± 0.04 (50 mM PEL) to 4.06 ± 0.04 (30 mM + OL and 50 mM + OL PEL; Table 1). Because the pH values were well below the pKa of PEL, the acid was present mainly in the efficacious, undissociated form. While water pH will still need monitoring in postharvest wash systems using PEL-based sanitizers, especially in areas with high water pH, pH management will likely be less rigorous than is needed in chlorine-based systems.

Organic loading with grape tomato puree did not significantly affect the efficacy of any of the four sanitizers. This is contrary to previous studies which found that chlorine efficacy is greatly decreased in the presence of organic material. Dunn et al. [14] found that a 1% (*w*/*v*) sweet pepper puree significantly decreased chlorine efficacy against *Salmonella* on inoculated peppers and in postharvest wash. The pepper study also found that *Salmonella* levels recovered from a used chlorine wash solution supplemented with 1% organic load were not significantly different from levels recovered from used water. In another study, organic loading using <2.5% blended iceberg lettuce reduced chlorine efficacy against *E. coli* O157:H7 [48]. Shen et al. found that the addition of <0.3% tomato extract rapidly depleted free chlorine, while significantly increasing chemical oxygen demand and turbidity [49]. PAA was also not significantly impacted by organic loading, in agreement with a study that found that the efficacy of 50 ppm PAA against *E. coli* O157:H7 was not significantly affected up to 10% organic load in the form of blended iceberg lettuce [50]. Finally, neither PEL treatment was affected by organic loading, which was anticipated as organic acids are highly stable in the presence of organic material [12]. These studies indicate that the 0.5% organic load supplemented in the current study was likely insufficient to quench the antimicrobial activity of any of the examined sanitizers, including chlorine; a higher level (>0.5%) of organic loading may have elicited a significant reduction in efficacy. The brix of the tomato puree was not measured in the current study but this information could have provided insight into the effectiveness of the organic loading methodology. Tomato extract or sterilized soil, instead of tomato puree, may have been more suitable proxies for simulated organic loading in a produce wash system.

Firmness and skin strength of the grape tomatoes treated with water, chlorine and PAA, as well as NR, remained relatively stable (no significant changes) during storage. Fan et al. [51] also found that the firmness of grape tomatoes after rinsing with water remained constant throughout 21 d storage at 10 °C. Chlorine is known to have minimal negative effects on produce quality parameters such as texture [35]. Vandekinderen et al. [52] conducted triangle sensory tests comparing the effects of water and various sanitizers on fresh-cut carrots (*n* = 18). There was a significant difference between carrots treated with water and 200 ppm chlorine; deviant taste and odor were noted by panelists. In the same study, there was not a significant difference between carrots treated with water versus those treated with 80 ppm PAA. However, there was a significant difference between carrots treated with water versus carrots treated with 250 ppm PAA; deviant taste and texture were noted.

A disadvantage of PEL as a produce wash can likely be attributed to its activity against cell membranes of plant and microbial cells alike. Pelargonic acid desiccates plant tissues by disrupting the cell membrane and removing the waxy cuticle, which results in cell leakage and rapid cell death similar to that seen in prokaryotic cells, leading to tissue discoloration and loss of structural integrity [24]. Exposure to 50 mM fumaric acid for 10 min reduced *S. enterica* ser. Typhimurium, *Staphylococcus aureus and E. coli* O157:H7 populations on lettuce by over one log; however, tissue browning occurred [53]. In this study, grape tomatoes exposed to PEL did not exhibit noticeable color change but significant textural changes occurred after 1 d of storage. Both firmness and skin strength decreased significantly from 0 d to 1 d for tomatoes that were treated with PEL. Decreased firmness and skin strength in tomatoes treated with PEL were expected, as PEL can penetrate the waxy cuticle and disrupt membrane permeability. These actions lead to cell leakage and desiccation, similar to the effect seen on PEL-exposed bacteria [22,24,54]. Due to chemical and structural differences, it is possible that other tomato cultivars or stages of maturity may be more tolerant to the phytotoxic activity of PEL [55,56,57]. Incorporation into polysaccharide films, such as pullulan and chitosan, has shown promise for other antimicrobials used on fresh produce and may be a useful delivery mechanism for PEL-based sanitizers [58]. Aside from the impact of PEL on texture, another consideration is that PEL has a fatty, coconut aroma, which in some cases has been described as rancid [59,60]. In this study, a coconut aroma was noticeable and persisted from the time of treatment through 7 d of tomato storage, which is undesirable for fresh produce.

## 5. Conclusions

While an effective biocidal agent against *Salmonella* in a simulated wash system, the current PEL formulation is not a likely candidate to replace chlorine or PAA as a postharvest wash due to its negative organoleptic impact on the tomato surface. However, the current formulation could have promise as a sanitizer for food contact or non-food contact surfaces; not only is it efficacious against *Salmonella*, but it may also be a safe alternative for use around food in a packing or production facility that has increased consumer acceptability due to its formulation. While reformulation may be necessary before consideration as a postharvest sanitizer, the ability to control against *Salmonella* contamination, as well as the long-term antagonistic activity exerted by PEL at both concentrations suggests that continued development of PEL-based sanitizers could eventually yield promising sanitizer alternatives for the food industry.

## Figures and Tables

**Table 1:** Mean pH ± standard deviation (*n* = 12) of wash treatments.

Treatment	Mean pH
PAA	3.71 ± 0.05
PAA + OL	3.85 ± 0.06
30 mM PEL	3.91 ± 0.04
30 mM PEL + OL	4.06 ± 0.04
50 mM PEL	3.89 ± 0.04
50mM PEL + OL	4.06 ± 0.04
Chlorine	7.00 ± 0.02 ^1^
Chlorine + OL	7.00 ± 0.02 ^1^

^1^ final pH after adjustment with citric acid. PAA, peroxyacetic acid; OL, organic load; PEL, pelargonic acid.

**Table 2:** Log populations (CFU/g) of *Salmonella enterica* on the inoculated tomato surface immediately after sanitizer treatment (0 d) and throughout 1 d and 7 d storage at 8 °C. There were no significant third and second order interactions among organic load (OL), sanitizer and storage duration treatments, so OL and non-OL sanitizer data were analyzed and reported together (sanitizer *n* = 16; no treatment *n* = 8).

	Storage Time (d)					
	0		1		7	
	Log CFU/g					
Sanitizer						
Chlorine	7.03 ± 0.06	a ^1^A ^2^	5.38 ± 0.30	aB	5.28 ± 0.26	aB
PAA	6.73 ± 0.10	aA	3.23 ± 0.51	bB	2.92 ± 0.50	bB
30 mM PEL	5.76 ± 0.08	bA	1.86 ± 0.21	cB	1.00 ± 0.00	cC
50 mM PEL	5.37 ± 0.17	bA	1.16 ± 0.08	cB	1.15 ± 0.10	cB
No treatment						
Water	7.35 ± 0.05		7.59 ± 0.06		7.57 ± 0.11	
NR	7.53 ± 0.11		7.96 ± 0.14		7.73 ± 0.22	
Contrasts						
Sanitizer vs. Water						
Chlorine × Water	ns		**		**	
PAA × Water	ns		***		***	
30 mM PEL × Water	**		***		***	
50 mM PEL × Water	**		***		***	
Sanitizer vs. NR						
Chlorine × NR	ns		**		**	
PAA × NR	ns		***		***	
30 mM PEL × NR	**		***		***	
50 mM PEL × NR	**		***		***	
No treatment						
Water × NR	ns		ns		ns	

^1^ Values followed by similar lowercase letter indicate no significant differences (*p* ≥ 0.05) among sanitizer treatments (rows) within storage time (columns). ^2^ Values followed by similar uppercase letter indicate no significant differences (*p* ≥ 0.05) among storage time (columns) within sanitizer treatments (rows). ns, **, *** nonsignificant or significant at *p* ≤ 0.01, 0.001, respectively. PAA, peroxyacetic acid; OL, organic load; PEL, pelargonic acid; NR, no rinse.

**Table 3:** Effect of the interactions among sanitizer treatments (including water and no rinse (NR) controls) and storage time on tomato skin penetration (N; *n* = 8).

Treatments	Storage Time (d)					
	0		1		7	
	Force (N)					
Chlorine	9.04 ± 0.55	ab ^1^A ^2^	9.00 ± 0.45	aA	8.68 ± 0.69	aA
PAA	8.81 ± 0.66	abA	9.85 ± 0.66	aA	8.41 ± 0.45	aA
30 mM PEL	8.51 ± 0.83	abA	4.68 ± 0.19	bB	3.94 ± 0.15	bB
50 mM PEL	7.23 ± 0.29	bA	4.61 ± 0.34	bB	3.51 ± 0.21	bB
Water	9.02 ± 0.48	abA	9.93 ± 0.57	aA	9.21 ± 0.40	aA
NR	9.82 ± 0.72	aA	9.80 ± 0.49	aA	8.68 ± 0.59	aA

^1^ Values followed by similar lowercase letter indicate no significant differences (*p* ≥ 0.05) among treatments (rows) within storage time (columns). ^2^ Values followed by similar uppercase letter indicate no significant differences (*p* ≥ 0.05) among storage time (columns) within treatments (rows). PAA, peroxyacetic acid; PEL, pelargonic acid; NR, no rinse.

**Table 4:** Effect of the interactions among sanitizer treatments (including water and NR controls) and storage time on tomato surface compression (N; *n* = 8).

Treatments	Storage Time (d)					
	0		1		7	
	Force (N)					
Chlorine	15.47 ± 0.84	a ^1^A ^2^	16.24 ± 1.30	aA	18.80 ± 0.78	aA
PAA	16.87 ± 1.30	aB	15.29 ± 0.72	aB	19.46 ± 1.58	aA
30 mM PEL	17.27 ± 1.06	aA	7.01 ± 0.29	bB	8.60 ± 0.49	bB
50 mM PEL	15.30 ± 1.69	aA	7.17 ± 0.54	bB	6.14 ± 0.33	bB
Water	16.17 ± 0.63	aB	16.45 ± 1.16	aB	20.54 ± 1.50	aA
NR	15.82 ± 1.09	aB	16.47 ± 0.63	aB	19.99 ± 1.09	aA

^1^ Values followed by similar lowercase letter indicate no significant differences (*p* ≥ 0.05) among treatments (rows) within storage time (columns). ^2^ Values followed by similar uppercase letter indicate no significant differences (*p* ≥ 0.05) among storage time (columns) within treatments (rows). PAA, peroxyacetic acid; PEL, pelargonic acid; NR, no rinse.
